# Supplementary material for: Expression and Prognostic Characteristics of Ferroptosis-Related Genes in Colon Cancer
Source: Int J Mol Sci. 2021 May 26;22(11):5652. doi: 10.3390/ijms22115652 (PMC8199073; doi:10.3390/ijms22115652)
Supplement: Supplementary file 1 [file ijms-22-05652-s001.zip › Supplementary Materials_final version.pdf]

Supplementary Materials:

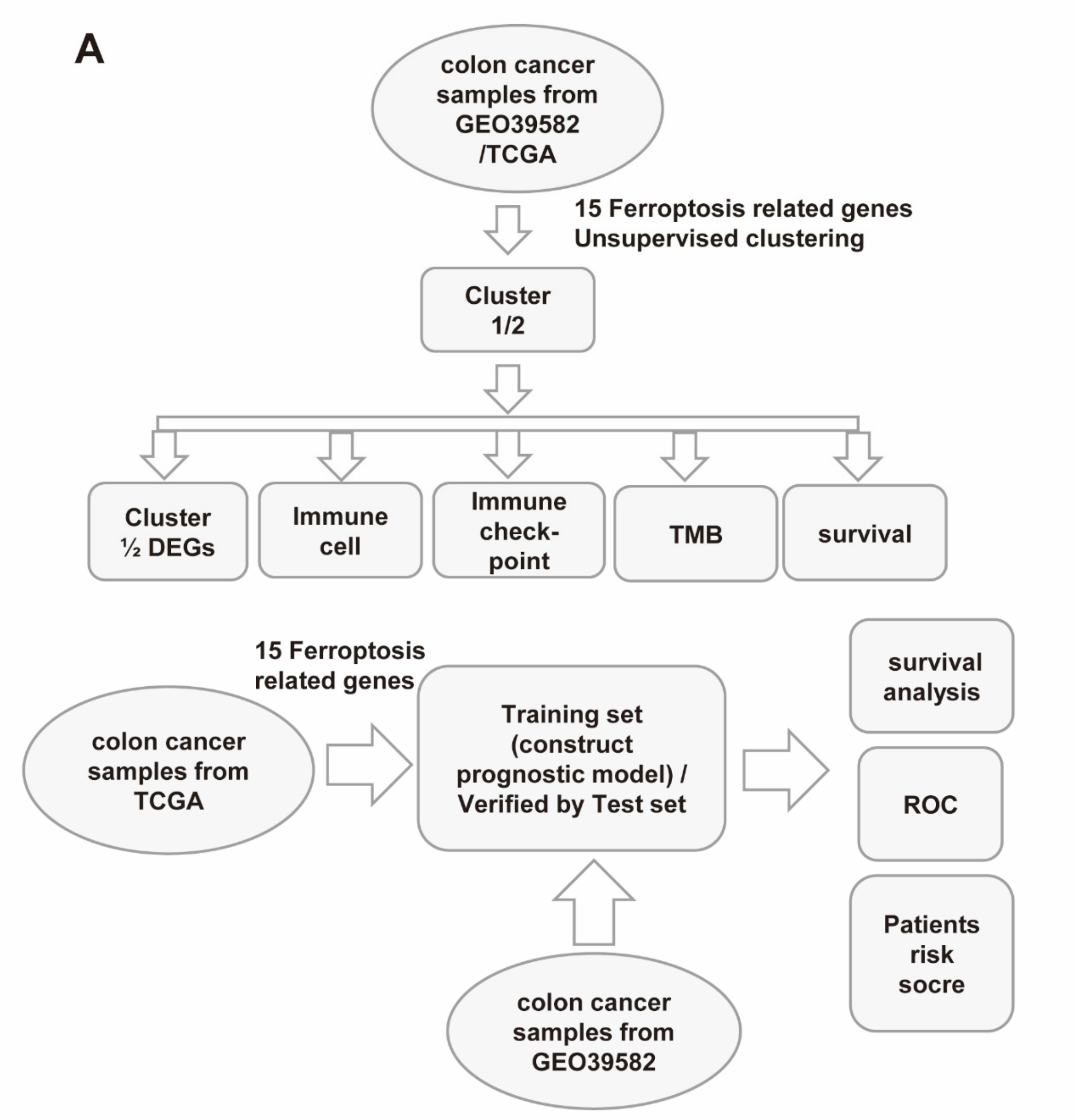

S1: Overview of study design. (A) Overview of this work.

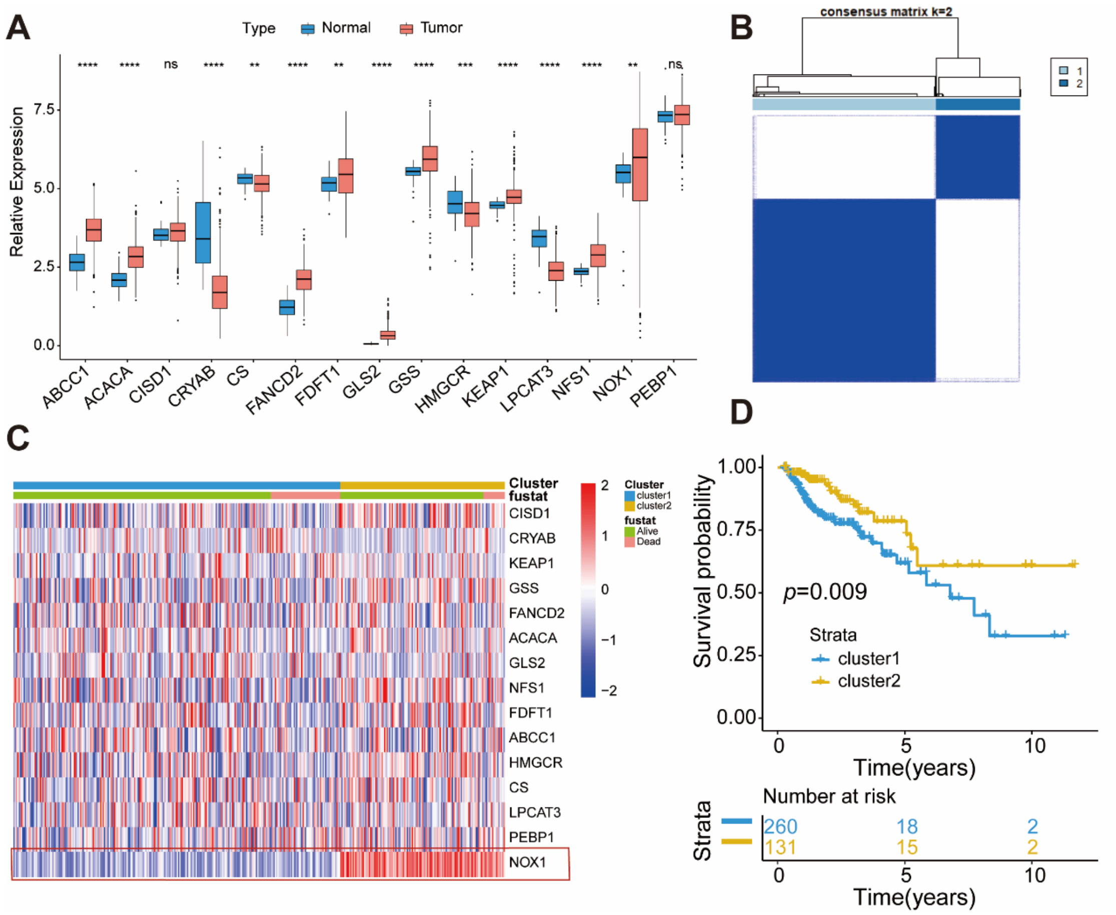

S2: The characteristics of two molecular subgroups of colon cancer.

(A) The expression of 15 ferroptosis-related genes between normal tissues and colon cancer tissues in TCGA. Tumor, red; Normal, blue. The upper and lower ends of the boxes represented interquartile range of values. The lines in the boxes represented median value, and the black dots showed outliers. The asterisks represented the statistical *p* value (***p*<0.01, ****p*<0.001, *****p*<0.0001, ns means no significance). (B) The consensus matrices of the TCGA for k=2. Survival analyses for two molecular subgroups of colon cancer based on 562 patients with colon cancer from GEO cohorts (GSE39582) including 188 cases in cluster1, 374 cases in cluster2. (C) Unsupervised clustering of 15 ferroptosis-related genes in the GSE39582. The cluster and status were used as patient annotation. Red represented high expression of regulators and blue represented low expression. (D) Kaplan-Meier curves with Log-rank *p* value 0.009 showed a significant survival difference within two molecular subgroups of colon cancer.

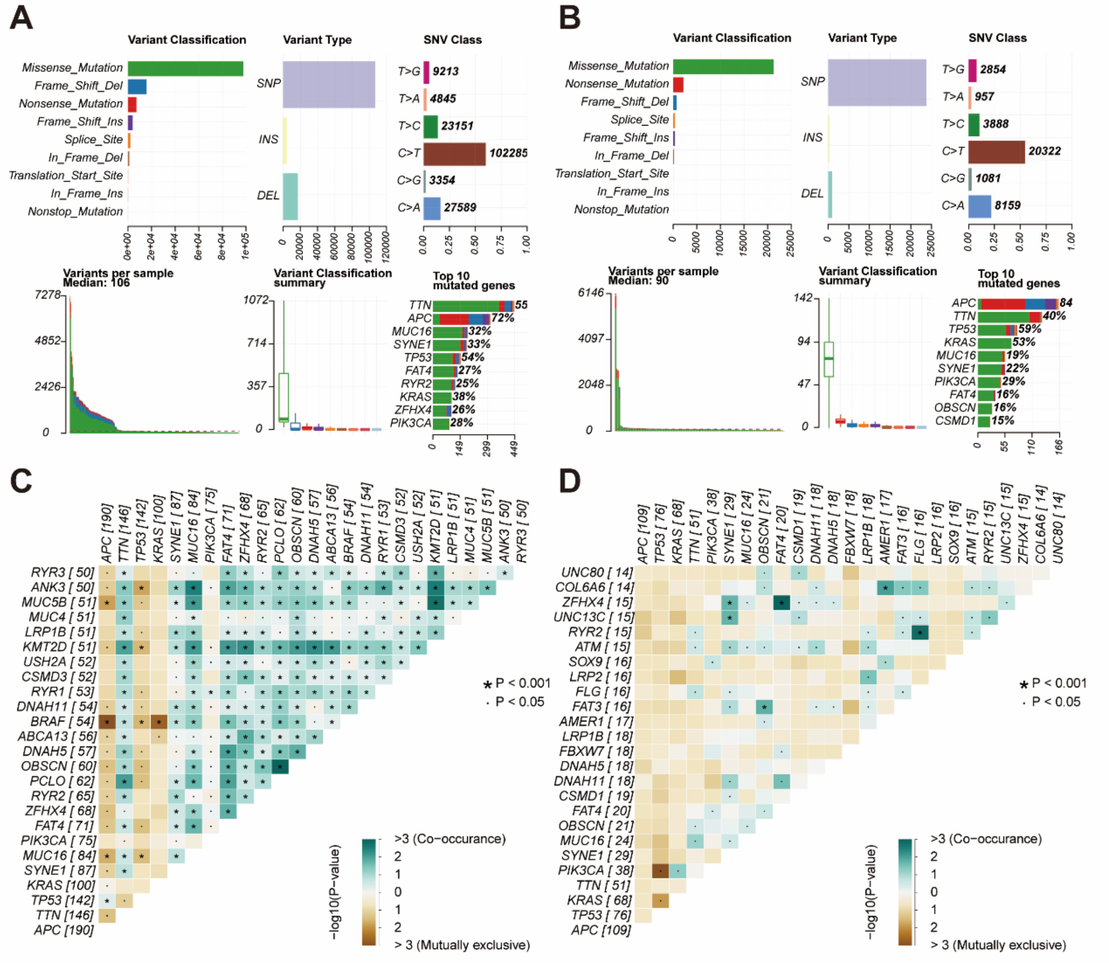

S3: The TMB of two cluster. (A-B) The summary mutation of cluster1 (A) and cluster2 (B). (C-D) The gene mutation interaction of cluster1 (C) and cluster2 (D).

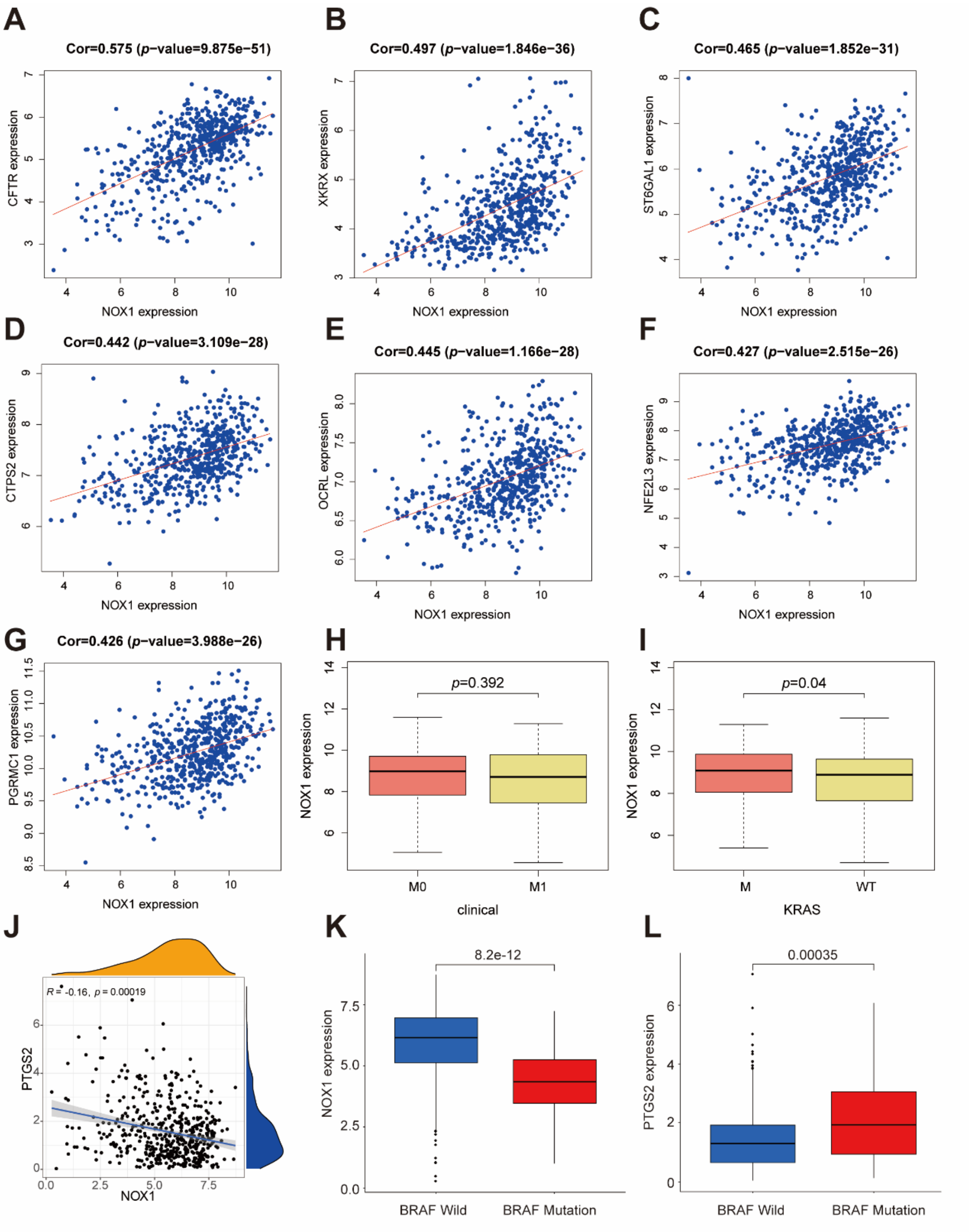

S4: The genes and clinical feature related to *NOX1*. (A-G) The ~~cor~~relationship between *CFTR*, *XKRK*, *ST6GAL1*, *CTPS2*, *OCRL*, *NFE2L3*, *PGRMC1* and *NOX1*. (J-L) The ~~co~~relationship between NOX1 and COX2 (PTGS2) (J), NOX1 and BRAF mutation (K), PTGS2 and BRAF mutation (L).

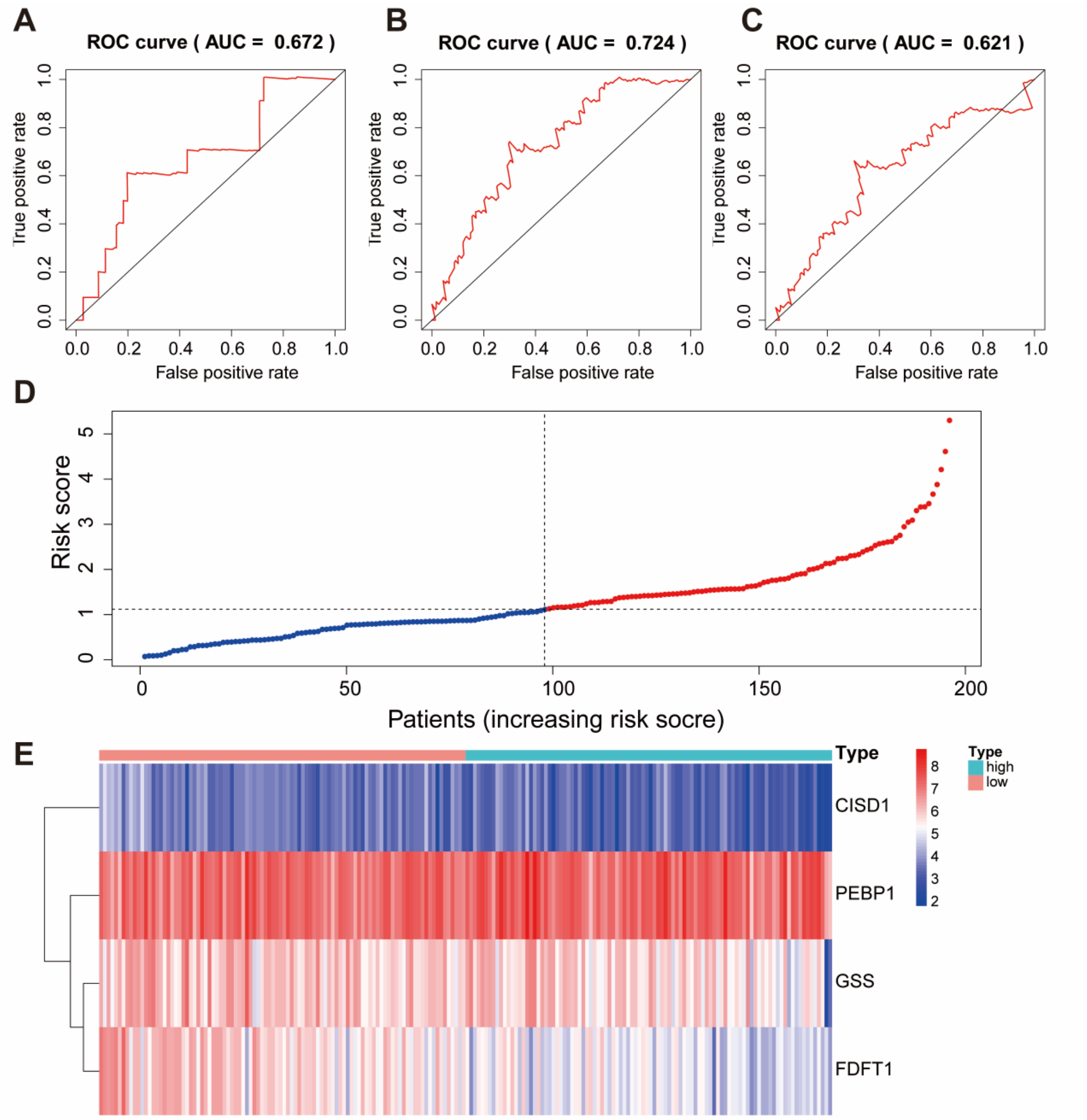

S5: Cox regression model results in training set of TCGA. (A-C) ROC curve of the ferroptosis risk score forecast result after 1, 3 and 5 year in training set of TCGA. (D) The distribution and median value of the risk score in the training set of TCGA. (E) The *PEBP1*, *CISD1*, *GSS* and *FDFT1* expression in high or low risk score group in the training set of TCGA.

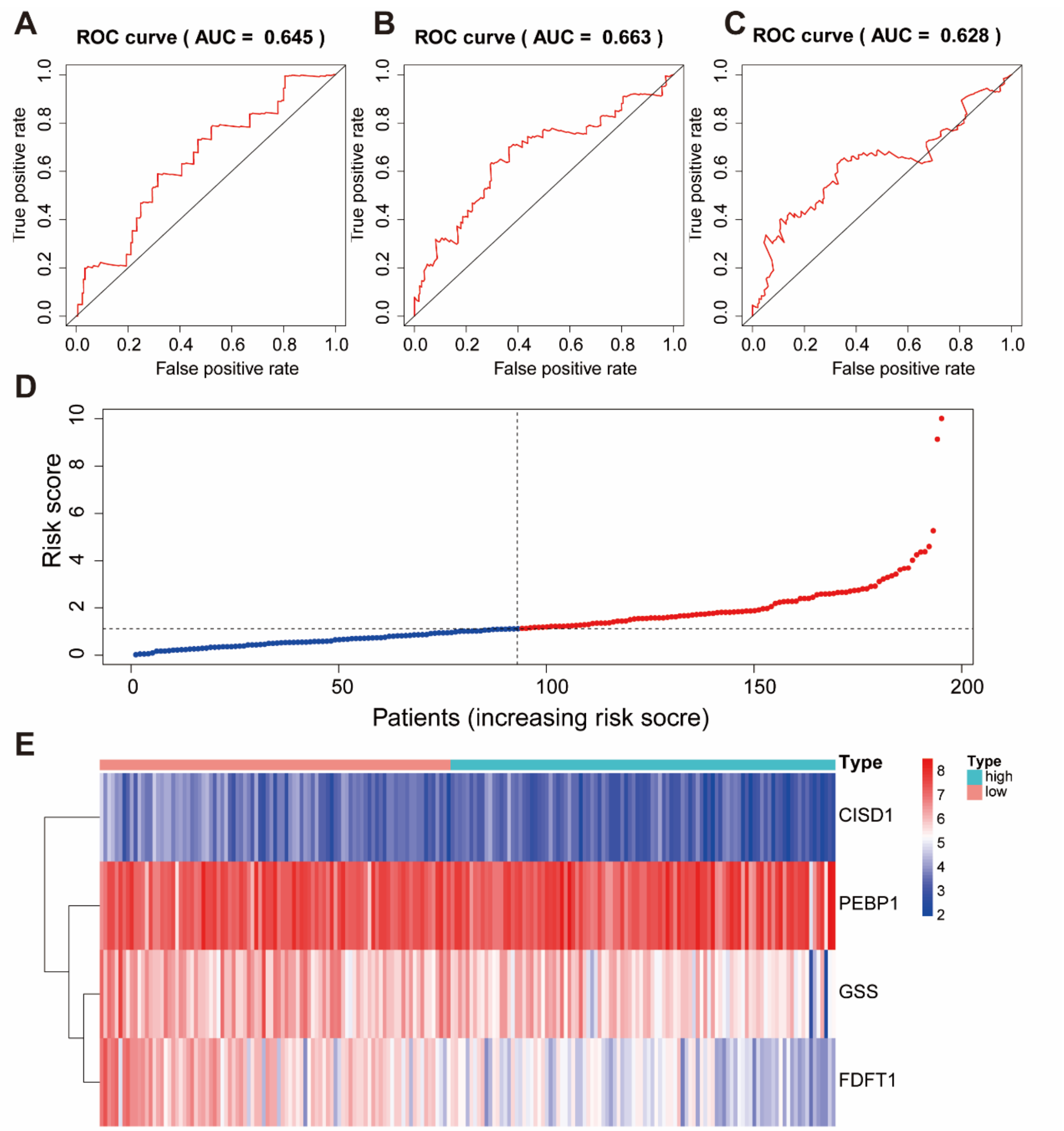

S6: Cox regression model results in test set of TCGA. (A-C) ROC curve of the ferroptosis risk score forecast result after 1, 3 and 5 year in test set of TCGA. (D) The distribution and median value of the risk score in the test set of TCGA. (E) The *PEBP1*, *CISD1*, *GSS* and *FDFT1* expression in high or low risk score group in the test set of TCGA.
